# Macrophage Activation Syndrome: A Report of Two Cases and a Literature Review

**DOI:** 10.1155/2017/5304180

**Published:** 2017-10-25

**Authors:** Asaad Alkoht, Ibrahem Hanafi, Basheer Khalil

**Affiliations:** ^1^Division of Rheumatology, Department of Internal Medicine, Faculty of Medicine, Damascus University, Damascus, Syria; ^2^Faculty of Medicine, Damascus University, Damascus, Syria; ^3^Division of Rheumatology, Department of Pediatrics, Faculty of Medicine, Damascus University, Damascus, Syria

## Abstract

Macrophage activation syndrome (MAS) is a severe, potentially fatal condition that may complicate autoimmune diseases, and it belongs to hemophagocytic lymphohistiocytosis (HLH) disorders. MAS occurs in adults and children. However, it is rare in juvenile systemic lupus erythematosus (jSLE), and it is extremely rare to be the initial presentation of jSLE. Here, we report two patients with juvenile SLE who initially presented with MAS. One of the two patients is 4 years old. This is the youngest reported patient to our knowledge.

## 1. Background

Macrophage activation syndrome (MAS) is a rare, severe, and potentially fatal condition that may complicate autoimmune diseases, and it belongs to a group of disorders called hemophagocytic lymphohistiocytosis (HLH) [1]. Basically, HLH has two forms: familial, which has an autosomal recessive inheritance pattern, and secondary, which is triggered by drugs, malignancies, infections, and rheumatic diseases [2]. Usually, MAS term is used to describe the secondary HLH triggered by rheumatic diseases as their initial manifestation or in the context of their course [3]. MAS occurs in both adults and children, and it has been reported with systemic juvenile idiopathic arthritis (sJIA), Still's disease, systemic lupus erythematosus, Kawasaki disease, and many other diseases [4]. The incidence of MAS in SLE patients is estimated to be 0.9% to 4.6% [2, 5], with a high mortality rate that reaches 8% to 22% [3, 6]. It has been suggested that MAS in juvenile SLE patients may be underrecognized and underreported [7]. To our knowledge, less than 20 cases of juvenile SLE that initially presented as MAS have been reported to date [8–13]. In this report, we describe two cases with their clinical manifestations, laboratory data, and management. One of the two patients is 4 years old; she is the youngest reported patient with juvenile SLE manifesting as MAS ever.

## 2. Case One

**Figure 1:**
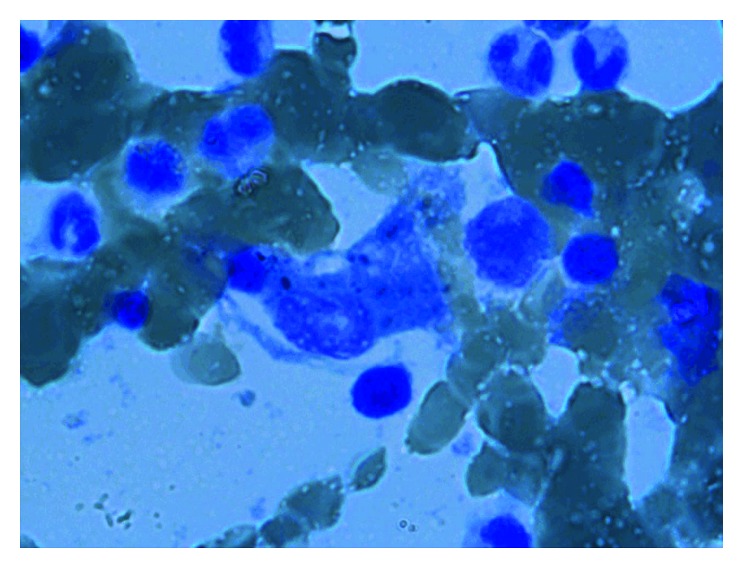
Bone marrow aspiration for case one showing macrophage activation syndrome (MAS).

A 9-year-old girl presented to Children's Hospital of Damascus University with a three-month history of abdominal pain, arthralgia, recurrent painless oral ulcers, bruises, epistaxis, anorexia, weight loss, night sweats, and fever. These complaints were preceded by agitation, aggression, and occipital headache that led her parents to ask for a psychiatric consult. She had no relevant past medical or travel history and no family history of rheumatic diseases. On physical examination, she had normal vital signs except for a temperature of 40°C. There was bilateral neck lymphadenopathy (1 × 1.5 cm) with splenomegaly and hepatomegaly. Laboratory studies showed leukopenia (3600 cells/µL), normocytic anemia (7.8 g/dl), thrombocytopenia (78,000 cells/µL), hyperferritinemia (591 µg/L), hypertriglyceridemia (281 mg/dl), hyperfibrinogenemia (559 mg/dl), and increased levels of lactate dehydrogenase (606 IU/L). Her PTT was 31 seconds, and the INR was 1. The levels of blood minerals, albumin, urea, serum creatinine, alanine aminotransferase (ALT), aspartate aminotransferase (AST), and total bilirubin were within the normal range. Urinalysis was also normal. Serological tests were negative for CMV, HBV, HCV, EBV, and HIV; the tuberculin sensitivity test and blood culture were also negative. Immunologically, the patient was positive for ANA (1/320 homogenous), anti-dsDNA (240 IU/ml), and anti-Sm (91 IU/ml), and her serum C3 and C4 complement factors were low (33 mg/dl and 1.8 mg/dl, resp.). In addition, direct and indirect Coombs tests were positive. Ultrasound revealed a moderate hepatosplenomegaly, and bone marrow aspiration revealed hyperactivity with some morphologically benign macrophages with an evidence of hemophagocytosis (Figure 1()). The girl was diagnosed with SLE initially presented as MAS. Management consisted of intravenous methylprednisolone (30 mg/kg/day) for three consecutive days followed by prednisolone (1 mg/kg) with cyclophosphamide (750 mg) per month for 6 consecutive months. The patient improved by this management and remained well after one year of follow-up.

## 3. Case Two

**Figure 2:**
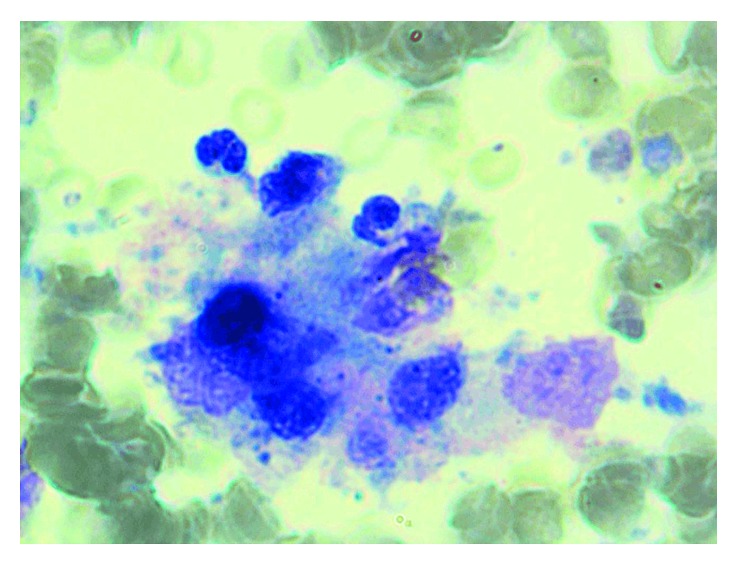
Bone marrow aspiration for case two showing macrophage activation syndrome (MAS).

A 4-year-old girl presented to Children's Hospital of Damascus University with a seven-day history of fever, dyspnea, constipation, and enlargement of the abdomen. On physical examination, she had a heart rate of 126 beats per minute, with a blood pressure of 120/90 mmHg, temperature of 39°C, and increased respiratory rate of 45 breaths/min. We also found splenomegaly, hepatomegaly, fine crackles in the right lung and reduced heart sounds, intercostal retraction, and increased abdominal circumference; however, the abdomen was soft and moving with breathing. Chest X-ray revealed enlarged heart, and ultrasound showed pericardial effusion. Laboratory data showed leukopenia (1900 cells/µL), microcytic anemia (8.2 g/dl), platelets of 173,000 cells/µL, erythrocyte sedimentation rate (ESR) of 85 mm/h1, elevated C reactive protein (CRP) of 2 mg/dl, hypoalbuminemia (2.6 g/dl), hyperfibrinogenemia (546 mg/dl), elevated lactate dehydrogenase (1279 IU/L), and hyperferritinemia (12,000 µg/L). Aspartate aminotransferase was elevated (163 IU/L), but alanine aminotransferase was normal (37 IU/L) as well as triglyceride, urea, creatinine, and blood minerals. Her PTT was 26 seconds, and the INR was 1. Urinalysis detected active urinary sediment with proteinuria (30 mg/dl), leukocyturia (14 WBCs/hpf), and hematuria (45 RBCs/hpf). Infection was ruled out with viral panel for CMV, HBV, HCV, EBV, and HIV; blood and urine cultures were negative. Immunologic studies were positive for ANA (1/160 homogeneous) and anti-dsDNA (1/160) but negative for anti-Sm. Serum C3 and C4 levels were low (54 mg/dl and 8 mg/dl, resp.). The direct and indirect Coombs tests were negative. Kidney biopsy was done, and it matched class IV (active and chronic) diffuse proliferative glomerulopathy according to the WHO classification [14]. Bone marrow showed a moderate decrease in cellularity, with 60% of the myeloid cells shifted to the left (less differentiated) with features of phagocytosis which led to the diagnosis of MAS (Figure 2()). During hospitalization, the patient developed tachycardia with generalized spastic convulsions. The patient was transferred to the intensive care unit where she had tracheal intubation and placed on mechanical ventilation. She underwent pericardial drainage, which yielded a yellow bloody fluid, while the biopsy of the pericardium showed chronic inflammatory reaction with negative culture for bacteria. The patient was given intravenous methylprednisolone (30 mg/kg/day) for 3 days followed by prednisolone (2 mg/kg/day) with cyclophosphamide (500 mg) per month for 6 consecutive months. The patient improved and remained well on after one year of follow-up.

## 4. Discussion

MAS is a hyperinflammatory state caused by proliferation and activation of T cells and macrophages, which produce an excessive inflammatory response and hypersecretion of cytokines such as interferon gamma (IFNγ), tumor necrosis factor (TNF), interleukin-1 (IL-1), IL-6, IL-10, IL-12, IL-18, and macrophage colony-stimulating factor [15]. MAS presents clinically with unremitting high fever, generalized lymphadenopathy, hepatosplenomegaly, and central nervous system involvement ranging in severity from mild confusion to seizures to frank coma. Patients develop hemorrhagic features resembling disseminated intravascular coagulopathy (DIC) and causing skin rashes and bleeding from the respiratory and gastrointestinal tract in more severe cases [1]. There are usually pancytopenia, elevation of serum liver enzymes, and abnormal coagulation profile with hypofibrinogenemia, hypertriglyceridemia, and hyperferritinemia [16]. Bone marrow examination shows numerous well-differentiated macrophages actively phagocytosing hematopoietic cells [16], and this usually confirms the diagnosis of MAS as in our two cases. However, that is not always possible due to sampling errors or infiltration of the macrophages in other tissues (e.g., liver, lymph nodes, skin, and lungs) or simply because the sampling is done early in disease course [1]. Although HLH has validated criteria for diagnosis by the International Histiocyte Society [17] (Table 1), MAS diagnosis is usually challenging, especially when it occurs in SLE patients who commonly have cytopenia, which cannot be distinguished from that of MAS [1].

Our two cases met the criteria of HLH with 6 criteria in case one and 5 criteria in case two, although two of the tests in these criteria were not available at our center: NK cell activity and serum sIL2Rα.

This raised the need to find diagnostic criteria for MAS in SLE patients. Parodi et al. [18] found that laboratory results are usually worse in MAS compared with SLE, except for white blood cell count and bilirubin, and their study highlighted the increase in CRP in MAS patients. Furthermore, they noticed that almost all MAS symptoms have high specificity and low sensitivity in diagnosing MAS except for fever, which has high sensitivity. The lab abnormalities that had the best sensitivity and specificity were hyperferritinemia, increased lactate dehydrogenase level, hypertriglyceridemia, and hypofibrinogenemia. Accordingly, the researchers suggested criteria for diagnosing MAS in juvenile SLE patients, and bone marrow aspiration was not considered necessary except in doubtful cases (Table 2). Our two cases met the criteria suggested by Parodi et al., with five clinical criteria and four laboratory criteria for case one and three clinical criteria and four laboratory criteria for case two. However, these criteria have some limitations. First, lab results were derived from only one center, and the study had a retrospective design with a small number of patients. Second, these criteria were suggested in order to differentiate MAS from active rheumatic diseases, which makes it difficult to distinguish MAS from some complications like infection.

Another tool to differentiate MAS from active rheumatic diseases was discussed again in a prospective study by Assari et al. [8] who studied the dynamic change and the lab value cutoff points of 17 juvenile MAS patients compared with 53 patients with active disease of SJIA, PJIA, Kawasaki disease, and SLE. Assari's study highlighted the decrease in platelets, liver function tests (AST and ALT), and albumin as the best laboratory data in the early diagnosis of MAS and its differentiation from the active rheumatic disease. This study also suggests that CRP may not help for the differentiation, and the cutoff point for ferritin was 5277 µg/L which is higher than that in the HLH criteria (500 ng/ml) [8].

Another multicenter retrospective cohort study [19] of 162 patients with reactive hemophagocytic syndrome suggested a new method for diagnosing MAS, called HScore (Table 3). This study was conducted in adult patients and may not be applicable to juvenile MAS patients [19]. However, when we applied it to our two patients, the probability of MAS was 88% and 99% in the order of our presentation.

While no randomized controlled trials were conducted on MAS patients and all the evidence is derived from case reports and case series, treatment remains a challenge. After ruling out infections, which are common triggers for MAS, the main initial treatment is high-dose intravenous corticosteroids, mostly methylprednisolone (30 mg/kg) for three days followed by oral prednisolone (2-3 mg/kg) [20]. If MAS does not improve with corticosteroids, several drugs are used, including cyclosporine-A, which was effectively used in several case series as part of HLH-2004 protocol treatment, similar to etoposide, which is preserved for refractory cases due to its potential liver toxicity [3]. Cyclophosphamide, which was used in our two cases, is especially used in SLE patients based on its known effectiveness for severe SLE cases [15].

## 5. Conclusion

MAS in SLE patients is a life-threatening underrecognized condition, which can be found even in very young patients. Early diagnosis is important to save patients from its bad outcomes, which points to the need for validated diagnostic criteria for MAS in jSLE patients.

## Figures and Tables

**Table 1:** Diagnostic criteria for macrophage activation syndrome: HLH-2004—revised diagnostic guidelines for HLH10.

The diagnosis of HLH can be established if one of the two criteria below is met
(1) A molecular diagnosis consistent with HLH (i.e., reported mutations found in either PRF1 or MUNC13-4), or
(2) Diagnostic criteria for HLH are fulfilled (i.e., at least five of the eight criteria listed below are present)
** **(a) Persistent fever
** **(b) Splenomegaly
** **(c) Cytopenia (affecting ≥ 2 of 3 lineages in the peripheral blood)
** **(i) Hemoglobin < 90 g/L (in infants < 4 weeks: <100 g/L)
** **(ii) Platelets < 100 × 10^9^/L
** **(iii) Neutrophils < 1.0 × 10^9^/L
** **(d) Hypertriglyceremia and/or hypofibrinogenemia
** **(i) Fasting triglycerides ≥ 3.0 mmol/L (i.e., ≥265 mg/dl)
** **(ii) Fibrinogen ≤ 1.5 g/L
** **(e) Hemophagocytosis in bone marrow^∗^ or spleen or lymph nodes, no evidence of malignancy
** **(f) Serum ferritin ≥ 500 µg/L (i.e., 500 ng/ml)
** **(g) Low or absent NK cell activity (according to local laboratory reference)
** **(h) Increased serum sIL2Rα (according to local laboratory reference)

^∗^If hemophagocytic activity is not proven at the time of presentation, further search for hemophagocytic activity is encouraged. If the bone marrow specimen is not conclusive, material may be obtained from other organs. Table 1 is copied from Henter et al. [17].

**Table 2:** Diagnostic criteria for macrophage activation syndrome: Parodi's preliminary diagnostic guidelines for MAS as a complication of juvenile SLE.

The diagnosis of MAS requires the simultaneous presence of at least 1 clinical criterion and at least 2 laboratory criteria
*Clinical criteria*
(1) Fever (>38°C)
(2) Hepatomegaly (≥3 cm below the costal arch)
(3) Splenomegaly (≥3 cm below the costal arch)
(4) Hemorrhagic manifestations (purpura, easy bruising, or mucosal bleeding)
(5) Central nervous system dysfunction (irritability, disorientation, lethargy, headache, seizures, or coma)
*Laboratory criteria*
(1) Cytopenia affecting 2 or more cell lineages (white blood cell count ≤ 4.0 × 10^9^/L, hemoglobin ≤ 90 g/L, or platelet count ≤ 150 × 10^9^/L)
(2) Increased aspartate aminotransferase (>40 U/L)
(3) Increased lactate dehydrogenase (>567 U/L)
(4) Hypofibrinogenemia (fibrinogen ≤ 1.5 g/L)
(5) Hypertriglyceridemia (triglycerides > 178 mg/dl)
(6) Hyperferritinemia (ferritin > 500 µg/L)
*Histopathologic criterion*
Evidence of macrophage hemophagocytosis in the bone marrow aspirate^∗^

^∗^Bone marrow aspiration for evidence of macrophage hemophagocytosis may be required only in doubtful cases. Table 2 is copied from Parodi et al. [18].

**Table 3:** Diagnostic criteria for macrophage activation syndrome: the HScore.

The diagnosis of MAS requires calculating the HScore	
(1) Known underlying immunosuppression^∗^: 0 (no) or 18 (yes)	
(2) Temperature (°C): 0 (<38.4), 33 (38.4–39.4), or 49 (>39.4)	
(3) Organomegaly: 0 (no), 23 (hepatomegaly or splenomegaly), or 38 (hepatomegaly and splenomegaly)	
(4) No. of cytopenias^†^: 0 (1 lineage), 24 (2 lineages), or 34 (3 lineages)	
(5) Ferritin (ng/ml): 0 (<2000), 35 (2000–6000), or 50 (>6000)	
(6) Triglyceride (mmoles/L): 0 (<1.5), 44 (1.5–4), or 64 (>4)	
(7) Fibrinogen (g/L): 0 (>2.5) or 30 (≤2.5)	
(8) Serum glutamic oxaloacetic transaminase (IU/L): 0 (<30) or 19 (≥30)	
(9) Hemophagocytosis features on bone marrow aspirate: 0 (no) or 35 (yes)	
Then, measure the assigned probability^‡^	

HScore	Probability of hemophagocytic syndrome, %

90	<1
100	1
110	3
120	5
130	9
140	16
150	25
160	40
170	54
180	70
190	80
200	88
210	93
220	96
230	98
240	99
250	>99

^∗^Human immunodeficiency virus positive or receiving long-term immunosuppressive therapy (i.e., glucocorticoids, cyclosporine, and azathioprine). ^†^Defined as a hemoglobin level of ≤9.2 g/dl and/or a leukocyte count of ≤5000 cells/µL and/or a platelet count of ≤110,000 cells/µL. ^‡^The best cutoff value for HScore was 169, corresponding to a sensitivity of 93%, a specificity of 86%, and accurate classification of 90% of the patients. Table 3 is copied from Fardet et al. [19].
